# Transcriptomic and Metabolomic Insights into the Enhanced Quality of *Anoectochilus roxburghii* Seedlings in Sugar-Free Versus Conventional Tissue Culture Systems

**DOI:** 10.3390/metabo16060374

**Published:** 2026-05-29

**Authors:** Chuanzhi Kang, Tongwei Lin, Hongyang Wang, Yiheng Wang, Dehua Wu, Wanying Duan, Zekun Zhang, Chengcai Zhang, Xiangtao Chen, Fangfang Chen

**Affiliations:** 1 State Key Laboratory for Quality Ensurance and Sustainable Use of Dao-di Herbs, National Resource Center for Chinese Materia Medica, China Academy of Chinese Medical Sciences, Beijing 100700, China; 2 Lishui Jianlan Biotechnology Co., Ltd., Lishui 323500, China13567181924@163.com (X.C.); 3 Hangzhou Mumu Biotechnology Co., Ltd., Hangzhou 311300, China; 13685781751@163.com

**Keywords:** *Anoectochilus roxburghii*, cultivation methods, metabolome, sugar-free tissue culture, transcriptome

## Abstract

**Background/Objective:** *Anoectochilus roxburghii*, a high-value medicinal orchid, faces significant challenges in quality standardization during large-scale tissue culture due to a lack of understanding of the underlying molecular mechanisms. This study aimed to compare “Jianlan No.2” plantlets cultured under a conventional tissue culture system (CK) and a sugar-free tissue culture system (TD), to elucidate the phenotypic and molecular basis for quality improvement. **Methods:** A systematic comparison was conducted. Phenotypic traits of plantlets from both systems were measured. Integrated transcriptomic (RNA sequencing) and untargeted metabolomic analyses were employed to identify the molecular differences at the gene expression and metabolite accumulation levels. **Results:** TD-grown seedlings exhibited significantly superior growth characteristics, including greater plant height, higher rooting rate, and improved transplant survival. Transcriptomic analysis identified 416 differentially expressed genes (DEGs) (44 upregulated, 372 downregulated in TD), which were significantly enriched in pathways related to cell wall organization, apoplast, and photosynthesis. Sixteen key genes were pinpointed as closely associated with seedling growth and metabolic regulation. Metabolomic profiling revealed 502 differentially accumulated metabolites (DAMs), with significant perturbations primarily in phenylpropanoid biosynthesis and terpenoid metabolism. **Conclusions:** The sugar-free tissue culture system enhances *A. roxburghii* seedling quality by coordinately modulating photosynthetic capacity, carbon metabolism, and the biosynthesis of key secondary metabolites. These findings provide a crucial molecular foundation for optimizing tissue culture protocols and advancing the standardized, high-quality cultivation of this valuable medicinal plant.

## 1. Introduction

*Anoectochilus roxburghii* (Wall.) Lindl., a perennial herb of the Orchidaceae family, is of considerable importance in traditional medicine and modern horticulture because of its medicinal value and ornamental characteristics [1,2]. As a high-demand medicinal material, with dried products fetching prices exceeding USD 500 per kilogram in certain markets, reflecting its substantial economic potential, tissue culture serves as a core technique for large-scale production, which not only alleviates the scarcity of wild resources but also enables quality-oriented optimization through precise control of environmental conditions [3,4]. However, current micropropagation systems still face a critical limitation: the regulatory effects of culture conditions (e.g., light cycles, hormone ratios, and nutrient composition) on secondary metabolite accumulation remain unclear, resulting in substantial variation in active compound content, stress tolerance, and plantlet morphogenesis [5]. These discrepancies directly undermine the clinical efficacy and market competitiveness of medicinal plant materials, creating a major barrier to the standardization and sustainable development of the entire industry [6]. Accordingly, elucidating molecular regulatory networks at the multi-omics level (transcriptomics and metabolomics) has become essential for overcoming quality bottlenecks and enabling precision cultivation.

As a rare medicinal plant with a long history of folk medicinal use, *A. roxburghii*, which is widely propagated via tissue culture for seedling production, is used medicinally in its entirety and possesses effects such as clearing heat, cooling blood, eliminating dampness, detoxifying, calming the liver, and relieving convulsions, with clinical indications for hepatitis, pulmonary tuberculosis, diabetes, rheumatoid arthritis, snakebite poisoning, and other conditions [7,8]. In conventional tissue culture (CK), sucrose is routinely added to the medium as a carbon source. In contrast, sugar-free tissue culture (TD) has recently emerged as an alternative approach, establishing a photoautotrophic system through controlled gaseous and light environments [9]. These two cultivation systems differ markedly in physiological metabolism and secondary metabolite accumulation. However, the molecular mechanisms by which they influence the quality of *A. roxburghii* in vitro seedlings remain insufficiently characterized. CK relies on exogenous sucrose supply, representing a heterotrophic growth mode that, while providing rapid energy, may disrupt normal metabolic regulation. Excess sucrose alters carbon allocation, suppresses photosynthesis-related gene expression, and consequently affects secondary metabolite biosynthesis [10]. Compared with CK methods, TD restores photosynthetic autotrophic capacity by simulating natural light conditions, bringing cultivation closer to the plant’s natural growth state and better maintaining metabolic balance [11]. However, there remains a lack of systematic and mechanistic elucidation of how these two cultivation systems synergistically regulate the transcriptomic and metabolomic networks to influence the quality of *A. roxburghii* tissue-cultured seedlings. Specifically, the key molecular differences between the sugar-free tissue culture system and the conventional method in promoting photoautotrophy, reshaping carbon allocation, and regulating the biosynthetic pathways of secondary metabolites have not yet been clarified.

Previous studies have demonstrated that light intensity and quality affect the expression of phenylpropanoid pathway genes through photoreceptor-mediated signaling, thereby regulating the biosynthesis of active compounds such as flavonoids and phenolic acids [12]. Meanwhile, plant hormones (e.g., auxin and cytokinin) act synergistically in cell differentiation and metabolic reprogramming [13]. For example, methyl jasmonate treatment significantly increases flavonoid content in *A. roxburghii* by activating MYB transcription factors and upregulating downstream biosynthetic genes [14]. However, most existing studies have focused on single-factor effects and lack integrated multiomics analyses, limiting comprehensive characterization of the interaction network among environmental factors, gene expression, and metabolism. Furthermore, differences in metabolic profiles between tissue-cultured seedlings and wild-type plants suggest that tissue culture may induce epigenetic modifications or regulatory changes mediated by noncoding RNAs, further increasing mechanistic complexity. Multiomics approaches provide powerful tools for elucidating the mechanisms underlying plant quality formation [15,16,17]. Transcriptomics enables analysis of gene expression dynamics and can identify differentially expressed genes (DEGs) under varying culture conditions. Comparative transcriptomic analysis of TD and CK *A. roxburghii* seedlings can reveal key genes involved in carbon metabolism, photosynthesis, and secondary metabolism. These expression changes may directly influence metabolite synthesis and accumulation. For instance, upregulation of photosynthesis-related genes may enhance light energy utilization efficiency, thereby promoting secondary metabolite biosynthesis [18]. Concurrently, metabolomic analysis provides comprehensive profiling of metabolite composition and abundance, offering direct evidence of how culture methods influence plant quality [19].

Integrating transcriptomic and metabolomic datasets, this study aims to elucidate the molecular regulatory networks underpinning quality formation in *A. roxburghii* seedlings under different tissue culture systems. We hypothesize that the sugar-free (TD) system, by restoring photoautotrophic growth, will induce distinct transcriptional programs and metabolic shifts compared to conventional culture (CK). Specifically, we anticipate that TD conditions may enhance photosynthetic efficiency and carbon assimilation, leading to re-routed metabolic flux towards the biosynthesis of key bioactive compounds, such as flavonoids and terpenoids. Furthermore, through integrated analysis, we expect to identify critical genes that are co-expressed with the altered metabolite profiles, thereby constructing gene–metabolite association networks. These anticipated findings are expected to not only advance our understanding of how cultivation strategies fundamentally shape plant physiology and metabolism at the multi-omics level but also offer a scientific framework for optimizing tissue culture protocols. Ultimately, this work seeks to provide mechanistic insights that will inform and enable the standardized, high-quality production of *A. roxburghii* and potentially other medicinal plants.

## 2. Materials and Methods

### 2.1. Plant Materials

This experiment employed 30-day-old tissue-cultured plantlets of *A. roxburghii* “Jianlan No. 2” as research materials for a comparative study under two distinct cultivation systems, with the original explants being stem segments of wild *A. roxburghii* collected from Jiulong Mountain, Jiulong Township, Jingning County, Lishui City, Zhejiang Province (28.35° N, 118.87° E). Three independent biological replicates were established for each cultivation system, with each replicate consisting of 30 uniformly developed plantlets to minimize intra-group variation. The traditional tissue culture (CK) system was based on MS medium using stem segments with axillary buds as explants. The seeds were sterilized with 0.1% HgCl_2_ solution for 10 min, whereas the stem segments underwent sequential sterilization using 75% ethanol and 0.1% HgCl_2_. The medium was supplemented with 80 g/L banana homogenate and 1 g/L activated carbon. The hormone ratio during the cluster bud induction phase was 6-BA 2.0 mg/L combined with NAA 0.1 mg/L, which was adjusted to 6-BA 0.5 mg/L and NAA 0.5 mg/L upon transition to the seedling strengthening stage. The cultivation conditions included an initial 2-week dark period, followed by growth under 2000–3000 Lx light intensity, a 12 h photoperiod, and a constant temperature of 25 °C. The sugar-free box acclimation (TD) system replaced conventional sugar sources by introducing CO_2_ gas, promoting autotrophic growth in plants. This system utilized a mixed substrate of 10–30 mm Pindstrup peat and perlite, with 50 robust tissue-cultured plantlets (>6 cm tall) established per box. Before transplantation, plantlets were thoroughly washed to remove residual medium and disinfected using carbendazim. The cultivation setup comprised a CO_2_ cylinder, monitoring and control system, and gas delivery unit. Environmental parameters were maintained at 800–1600 ppm CO_2_, 25–26 °C temperature, and 3000–5000 Lx light intensity. During the initial phase, humidity was kept above 85% and then gradually reduced to promote acclimatization and enhance environmental adaptability.

### 2.2. RNA Extraction and Illumina Sequencing

Total RNA was extracted from tissue-cultured *A. roxburghii* seedlings using magnetic bead-based purification. Three biological replicates were included for each experimental group, with each replicate consisting of pooled tissue from three uniform, healthy seedlings to minimize individual variation. Briefly, the samples were flash-frozen in liquid nitrogen and ground into powder, followed by homogenization in TRIzol reagent (Thermo Fisher Scientific, Carlsbad, CA, USA). The lysate was then subjected to phase separation with chloroform, isopropanol precipitation, and ethanol washing to purify RNA, and the quality was assessed by NanoDrop 2000/8000 spectrophotometry (A260/A280 ratio 1.8–2.1) (Thermo Fisher Scientific, Waltham, MA, USA) and 5400 system integrity analysis (28S/18S ratio ≥ 0.9, RQN ≥ 8.9) (Thermo Fisher Scientific, Waltham, MA, USA). Poly(A)+ mRNA was enriched from 1 μg total RNA using oligo(dT)-coated magnetic beads (Yeasen, Shanghai, China), fragmented with fragmentation buffer at 94 °C, and reverse-transcribed into first-strand cDNA with random hexamers. Second-strand cDNA synthesis was then performed with DNA polymerase I and RNase H, followed by purification, end-repair, A-tailing, and Illumina adapter ligation. The library was amplified through 10–12 polymerase chain reaction (PCR) cycles, with fragment size distribution validated on the Agilent 2100 system (target peak ~350 bp) (Agilent Technologies, Santa Clara, CA, USA) before sequencing on the Illumina NovaSeq 6000 platform (Illumina, Inc., San Diego, CA, USA) (paired-end 150 bp), ensuring ≥6 Gb data per sample. This protocol adheres to rigorous quality control standards for transcriptome analysis [20].

### 2.3. Differential Gene Expression Analysis and Functional Annotation

Based on the MGI eukaryotic transcriptome sequencing data analysis pipeline, this study employed standardized bioinformatics methods: first, the filtered high-quality sequencing reads were aligned to the reference genome using HISAT2 v2.0.5, followed by gene expression quantification based on the FPKM method [21]. For differential expression analysis, DESeq2 software (DESeq2 package (version 1.44.0))was applied to samples with three biological replicates to identify DEGs using a negative binomial distribution model, with the Benjamini–Hochberg method used to correct *p*-values, whereas genes with adjusted *p*-values ≤ 0.05 were defined as significantly differentially expressed [22]. Functional annotation and enrichment analysis of the DEGs were performed using the clusterProfiler R package (4.12.6) for Gene Ontology (GO) functional enrichment and Kyoto Encyclopedia of Genes and Genomes (KEGG) pathway enrichment analysis, with a significance threshold of *p* < 0.05, systematically elucidating the biological functions and regulatory pathways of the DEGs.

### 2.4. Metabolite Extraction and Analysis

The metabolite extraction and analysis of tissue-cultured *A. roxburghii* seedlings followed a standardized untargeted metabolomics protocol. Three biological replicates were included for each experimental group, with each replicate prepared from pooled tissue of three uniform seedlings to ensure statistical robustness. For the dried samples, lyophilization was performed for 63 h, followed by grinding at 30 Hz for 1.5 min to obtain the powder. Precisely 30 mg of the sample was added to 1500 μL of pre-chilled (−20 °C) 70% methanol aqueous internal standard extraction solution (proportionally adjusted for samples < 30 mg) and vortexed for 30 s every 30 min (this step was repeated 6 times). The supernatant was centrifuged and filtered through a 0.22 μm microporous membrane. For fresh samples, liquid nitrogen freezing was applied before grinding for 30 s into powder, after which 50 mg of the sample was added to 600 μL of 70% methanol internal standard extraction solution, vortexed, and incubated at −20 °C for 30 min before centrifugation and filtration. Chromatographic separation was performed on the Waters ACQUITY UPLC HSS T3 column (Waters Corporation, Milford, MA, USA) (1.8 µm, 2.1 mm × 100 mm) at 40 °C with a flow rate of 0.40 mL/min, using mobile phase A (0.1% formic acid in water) and mobile phase B (0.1% formic acid in acetonitrile) with the following gradient elution: 0 min (95% A) → 2.0 min (75% A) → 4.0 min (1% A) → 4.5 min (1% A) → 4.6 min (95% A) → 6.0 min (95% A). Mass spectrometry detection was conducted on a Q Exactive HF-X mass spectrometer (Thermo Fisher Scientific, Waltham, MA, USA) with the electrospray ionization source in the positive/negative ion mode alternation, with spray voltages of 3500 V (+) and 3200 V (−), the ion transfer tube temperature at 320 °C, the nebulization temperature at 300 °C, a full scan range m/z of 84–1250, a resolution of 35,000, an automatic gain control of 1e6, a data-dependent MS2 scan resolution of 17,500, and collision energy settings of 30/40/50 V for three-stage fragmentation. Data preprocessing was used to convert raw data to mzML format via ProteoWizard (3.0.21229), followed by peak extraction, retention time correction, and peak alignment using XCMS (XCMS-V4.7). The missing values were imputed using KNN combined with 1/5 minimum value compensation, and the final quantitative data were obtained after support vector regression correction. The method ensured accurate and reproducible metabolite identification and quantification through rigorous quality control (QC), including QC sample total ion chromatogram (TIC) overlap analysis, correlation coefficient evaluation, and coefficient of variation (CV) value distribution validation.

Metabolite relative abundance was calculated based on the peak area. Significantly differential metabolites were identified by combining variable importance in projection (VIP) scores from partial least-squares discriminant analysis (PLS-DA) models with *p*-values from Student’s *t*-tests, using thresholds of VIP ≥ 1 and *p* < 0.05.

### 2.5. Selection of Key Differentially Expressed Genes

Integrated analysis of DEGs and differentially accumulated metabolites (DAMs) was performed by calculating Pearson’s correlation coefficients (PCCs) between the log2 fold change values of each metabolite and gene, using the R cor function. PCCs > 0.80 with a *p*-value < 0.05 were considered significant, revealing potential regulatory relationships between gene expression and metabolic phenotypes [23]. This approach combined transcriptomic and metabolomic data to identify the key associations underlying the biological responses of *A. roxburghii* tissue-cultured seedlings under different culture conditions.

From the pool of significantly differentially expressed genes (adjusted *p*-value ≤ 0.05), 16 key DEGs were selected based on the following criteria: high statistical significance (adjusted *p*-value < 0.01), expression fold change >2, and primary functional relevance to critical biological processes such as photosynthesis, stress response, chlorophyll biosynthesis, and cell-wall modification, which are central to the observed growth and metabolic differences between the conventional (CK) and sugar-free (TD) tissue culture systems. Furthermore, this selection process ensured that the chosen genes represent the most biologically meaningful regulators linking transcriptomic changes to the phenotypic and metabolic outcomes documented in the study.

### 2.6. Real-Time Quantitative PCR (RT-qPCR) Validation

RNA extraction and quality assessment were performed as a part of the RNA sequencing (RNA-Seq) workflow, followed by reverse transcription using the HiFi-MMLV cDNA First-Strand Synthesis Kit (Invitrogen, Thermo Fisher Scientific, Waltham, MA, USA). Three independent biological replicates were included for each experimental group, with each replicate derived from a distinct pool of uniform seedlings to account for biological variation. Six target genes were analyzed by RT-qPCR using primers designed with Primer Premier 5 (5.0) (Table S1), conducted on the ABI 7500 Fast Real-Time Detection System (Thermo Fisher Scientific, Waltham, MA, USA) (Applied Biosystems) with the Ultra SYBR Mix kit (CW BIO, Beijing, China) [24]. The reaction system (20 μL) included 10.4 μL of the Ultra SYBR Premix System II, 0.8 μL each of 10 μmol/L forward and reverse primers, 2 μL template, and sterile distilled water. The amplification protocol comprised a temperature of 95 °C for 10 min, followed by 40 cycles of treatment at 95 °C for 15 s and 55 °C for 1 min. Data were quantified using the 2^−ΔΔCT^ method with β-actin serving as the reference gene, and each sample was run in triplicate to ensure reproducibility.

### 2.7. Statistical Analysis

Statistical analyses were conducted using SPSS v.27.0 (SPSS Inc., Chicago, IL, USA). Data were presented as the mean ± SEM derived from three biological replicates. For the two-group comparison between cultivation treatments CK and TD, we employed an independent samples *t*-test, the statistically validated method for this experimental design [25]. Image analysis was performed using ImageJ software (version 1.53t, National Institutes of Health, Bethesda, MD, USA), and all figures were generated using OriginPro 2021.

## 3. Results

### 3.1. Effects of Different Culture Methods on Phenotypic Traits of A. roxburghii Plantlets In Vitro

**Figure 1:**
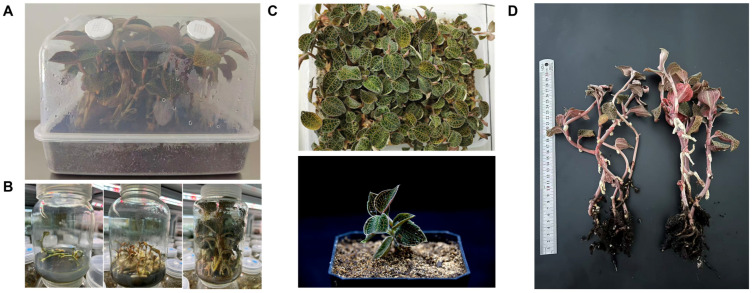
***A. roxburghii* plantlets under different culture methods**. (**A**) Sugar-free tissue culture of *A. roxburghii*; (**B**) conventional tissue culture of *A. roxburghii* (sterile line establishment—proliferation culture—rooting culture); (**C**) sugar-free in vitro plantlet transplantation (above) versus conventional in vitro plantlet transplantation (below); (**D**) comparison of sugar-free and conventional tissue culture methods for *A. roxburghii* plantlets. Panel 1C depicts the sugar-free tissue culture on the right and the conventional tissue culture on the left.

In this study, tissue-cultured plantlets of *A. roxburghii* “Jianlan No.2” were used to systematically compare the effects of CK and TD rooting methods on rooting capacity and growth performance. After 30 days of culture, aboveground growth parameters were measured. The results showed that TD plantlets exhibited significantly greater plant height (5.53 cm) (*p* ≤ 0.05), a higher rooting rate (87.35%), and a higher transplant survival rate (95.27%) than CK plantlets (Table 1). Morphological observations indicated that TD plantlets showed more vigorous growth, with larger leaves, fuller leaf morphology, and more uniform margins, whereas CK plantlets were smaller and exhibited leaf chlorosis in some cases (Figure 1).

### 3.2. Differential Gene Enrichment Analysis of Tissue-Cultured A. roxburghii Seedlings Under Different Culture Conditions

**Figure 2:**
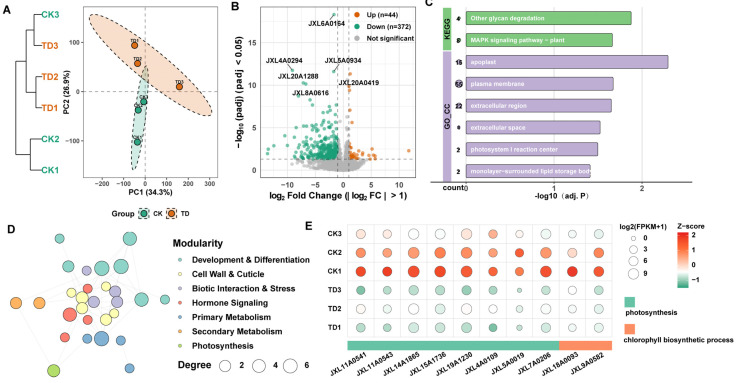
**Comparative transcriptomic analysis of *A. roxburghii* plantlets under different cultivation regimes.** (**A**) PCA of the two culture methods. The green (CK) and yellow (TD) circles represent traditional tissue culture and sugar-free tissue culture, respectively. To the left of the PCA is a dendrogram depicting the differences among samples of the two tissue culture methods. (**B**) Volcano map of differentially expressed genes (DEGs). (**C**) GO and KEGG enrichment analyses of DEGs. (**D**) GO_BP functional module enrichment network visualizing the enriched biological process (BP) GO terms categorized into seven functional classes: development and differentiation, cell wall and cuticle, biotic interaction and stress response, hormone signaling, primary metabolism, secondary metabolism, and photosynthesis. Nodes of different colors represent distinct functional modules, and node size (Degree) reflects the enrichment level, providing an intuitive representation of the multidimensional biological processes associated with DEGs. (**E**) High-expression GO_BP differential gene heatmap displaying the Z-score distribution of highly expressed genes in GO_BP categories such as photosynthesis and chlorophyll biosynthesis, illustrating their expression patterns across samples.

To elucidate the molecular mechanisms by which different cultivation methods affect the quality of *A. roxburghii* in vitro seedlings, this study analyzed seedlings produced using two distinct culture systems. Principal component analysis (PCA) showed that the CK group was tightly clustered along principal component 1 (34.3%) and principal component 2 (26.9%), while the TD system also formed a distinct cluster. A clear separation between the two groups was observed in the PCA score space, indicating that the TD system exerts a systematic regulatory effect on the gene expression profile of *A. roxburghii* (Figure 2A). Volcano plot analysis identified 44 significantly upregulated and 372 significantly downregulated genes (|log_2_FC| > 1, padj < 0.05). Among these, genes such as AR-HSP70-17 (JXL20A0419) and AR-PEL8 (JXL20A1288) showed particularly pronounced expression changes and high statistical significance (Figure 2B). GO functional enrichment analysis indicated that DEGs were primarily enriched in cellular components and regions such as the plasma membrane, extracellular region, and apoplast. KEGG enrichment analysis showed that DEGs were enriched in two pathways: plant MAPK signaling and other glycan degradation (Figure 2C).

To further characterize enriched biological processes, GO terms were subjected to modular analysis and classified into seven functional categories: development and differentiation, cell wall and cuticle, biotic interaction and stress, hormone signaling, primary metabolism, secondary metabolism, and photosynthesis. Genes associated with development and differentiation and with photosynthesis exhibited the most pronounced expression differences between the two groups (Figure 2D; Table S4). Heatmap analysis illustrated expression patterns of genes related to photosynthesis and chlorophyll biosynthesis across samples, showing that under TD conditions, several key photosynthesis-related genes in *A. roxburghii* were significantly downregulated (Figure 2E; Table S2).

### 3.3. Metabolomic Analysis and Metabolite Identification

**Figure 3:**
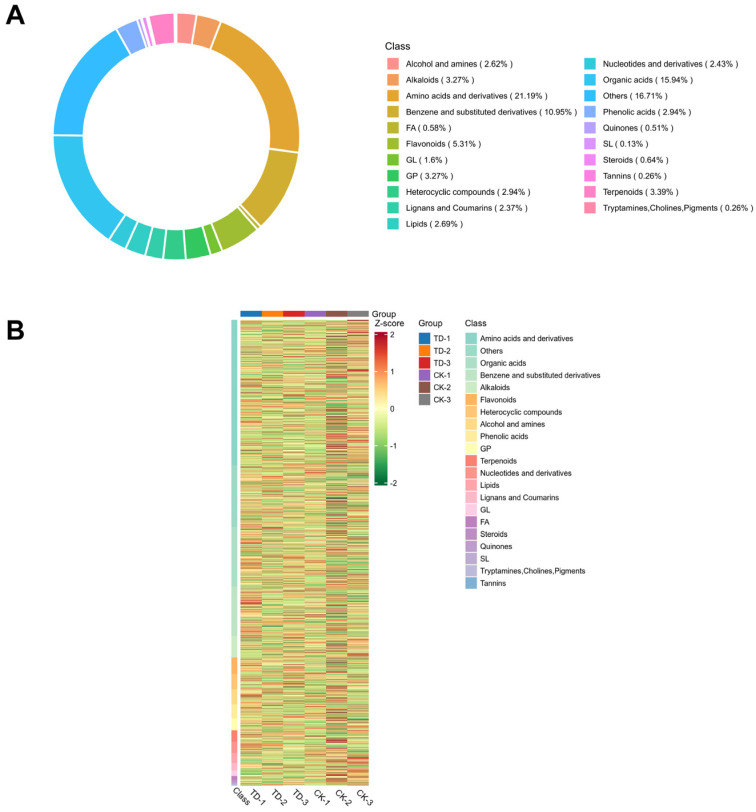
(**A**) Statistics of metabolites in all samples of *Anoectochilus roxburghii*. (**B**) Clustering heat map of all metabolites under different tissue culture systems, with metabolite data normalized using the min–max scaling method (each sample represented by a column, each metabolite by a row; color intensity reflects relative abundance, green: low, red: high).

To better characterize the metabolic differences in *A. roxburghii* under different cultivation strategies, a widely targeted metabolomic analysis using liquid chromatography tandem mass spectrometry was performed on plantlets grown under two distinct tissue culture systems. In total, 2814 metabolites were identified (Table S3). Among these, amino acids and their derivatives (21.19%) and organic acids (15.94%) were the most abundant classes, together accounting for more than 37% of the total and representing the dominant metabolite categories in the samples. Flavonoids (5.31%), terpenoids (3.39%), alkaloids (3.27%), and glycerophospholipids (GP) (3.27%) were present at moderate levels, whereas fatty acids (0.58%), quinones (0.51%), and other classes were detected at relatively low levels (Figure 3A).

The metabolite class heatmap showed that amino acids and their derivatives, as well as organic acids, were consistently abundant across sample groups, consistent with their overall proportions. In contrast, alkaloids, flavonoids, and other metabolites exhibited distinct expression patterns between the TD and CK groups. Several metabolites were highly abundant in the TD group but present at low levels in the CK group, indicating clear metabolic differences between the two cultivation systems (Figure 3B).

### 3.4. Identification of the DAMs Between Two Tissue Culture Systems

**Figure 4:**
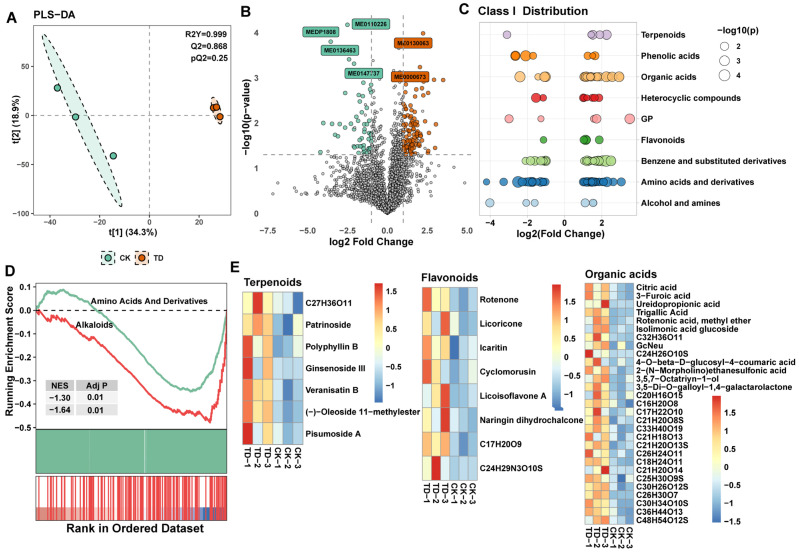
**Comparative metabolome analyses of *A. roxburghii* plantlets under different cultivation regimes.** (**A**) PLS-DA analysis plot. Partial least-squares discriminant analysis was employed to discriminate the samples, and the results demonstrated that the conventional tissue culture group (CK, green) and the sugar-free tissue culture group (TD, orange) exhibited completely separated clustering states in the score space. (**B**) Volcano plot of the 502 differentially expressed metabolites identified. (**C**) Heatmap of class I metabolite distribution. The X-axis represents log2 fold change, and different colors indicate different fold-change ranges. (**D**) Metabolite set enrichment analysis (MSEA) plot for amino acids and derivatives (NES = −1.30, Adj P = 0.01) and alkaloids (NES = −1.64, Adj P = 0.01). The Y-axis denotes the running enrichment score, the X-axis indicates the rank in the ordered dataset, and the bottom bar plot displays group distribution (CK: control group, TD: treatment group). NES reflects the enrichment degree, and Adj P denotes statistical significance after multiple testing corrections. (**E**) Heatmap of differential metabolites in terpenoids, flavonoids, and organic acids. The X-axis represents the sample group (CK/TD), and the Y-axis lists specific metabolites. Red indicates upregulation, blue indicates downregulation, and the color intensity corresponds to the magnitude of log2 fold change.

DAMs between sample pairs (CK vs. TD) were identified based on a VIP score of ≥1 and a fold change of ≥2 or ≤0.5. Metabolomic analysis was performed to characterize differences between the two tissue culture systems of *A. roxburghii*. The PLS-DA model (Figure 4A) showed strong model performance (R^2^Y = 0.999, Q^2^ = 0.888; *p* = 0.25), indicating clear separation and good predictive ability between the CK and TD groups, and suggesting distinct metabolic profiles associated with the two systems.

Volcano plot analysis further identified DAMs between the two culture systems. Among these, trans-zeatin (MEDP1808), (3-ethoxyphenyl-(2,3,4-trihydroxyphenyl) methanone (ME0000673), and four additional metabolites showed highly significant differences in accumulation, reflecting distinct metabolic regulation between the two systems (Figure 4B; Table S5). The class distribution plot (Figure 4C) indicated that terpenoids, phenolic acids, organic acids, and other metabolite classes exhibited varying degrees of change, with amino acids and their derivatives and alkaloids showing marked downregulation in the TD group.

Metabolite set enrichment analysis (Figure 4D) showed that amino acids and their derivatives (NES = −1.30, adj P = 0.01) and alkaloids (NES = −1.64, adj P = 0.01) were significantly enriched among downregulated metabolites in the TD group. The absolute NES values exceeded 1.3 and the adj P values indicated statistical significance, confirming global downregulation of these metabolite classes in TD. Heatmap analysis illustrated differential accumulation patterns of specific metabolites, including terpenoids (e.g., patrinoside and polyphyllin B), flavonoids (e.g., rotenone and icaritin), and organic acids (e.g., citric acid and 3furoic acid), with distinct patterns between the two groups, further supporting metabolic divergence between the two systems (Figure 4E; Table S5).

In this study, multiple DAMs were identified between the two tissue culture systems of *A*. *roxburghii*. Amino acids and their derivatives and alkaloids were the most prominently downregulated classes in the TD group, providing a metabolic basis for understanding how culture systems influence the metabolic profile of *A. roxburghii*.

### 3.5. Integrative Transcriptome and Metabolome Analysis Reveals Regulatory Networks Underlying Flavonoid and Terpenoid Biosynthesis

**Figure 5:**
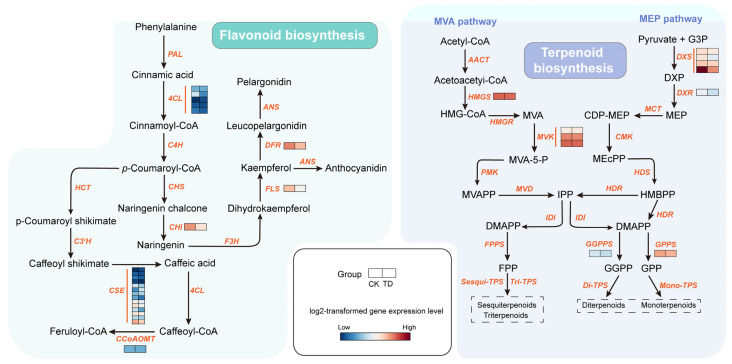
**Terpenoid and flavonoid biosynthesis pathways and their regulatory network in *A. roxburghii*.** Flavonoid biosynthesis pathway derived from phenylpropanoid metabolism. Terpenoid biosynthesis pathway, including the MVA and MEP pathways converging at IPP and DMAPP. The color scale represents relative gene expression levels under the conventional tissue culture group (CK) and the sugar-free tissue culture group (TD) conditions based on log2-transformed values, ranging from blue (low) to red (high).

In this study, an integrative transcriptome and metabolome analysis was conducted to compare the differences in flavonoid and terpenoid biosynthesis between sugar-free tissue culture (TD) and conventional tissue culture (CK) *A. roxburghii*. In the TD group, the expression of key genes in the flavonoid biosynthesis pathway, including PAL, 4CL, CSE, CCoAOMT, DFR, ANS, and FLS, was significantly upregulated, which promoted the enhanced synthesis of catechins, anthocyanins, and flavonols. In the terpenoid biosynthesis pathway, the expression of precursor synthesis genes in both the MVA pathway (HMGR, MVK) and the MEP pathway (DXR, HDR), as well as downstream terpenoid synthesis genes (Mono-TPS, Di-TPS, Tri-TPS), was upregulated, facilitating the accumulation of monoterpenoids, diterpenoids, and triterpenoids (Figure 5; Table S6). Overall, sugar-free tissue culture upregulated the core genes in both pathways, constructing a coordinately regulated metabolic network that significantly improved the synthesis efficiency of secondary metabolites in *A. roxburghii*.

### 3.6. RT-qPCR Validation of the Transcriptomic Data

**Figure 6:**
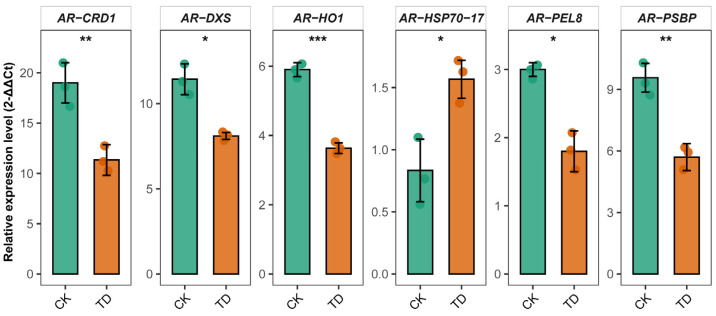
**RT-qPCR validation of representative gene expression in traditional tissue culture (CK) and sugar-free tissue culture (TD) of *A. roxburghii*.** Statistical significance of differences between the CK and TD groups was determined by *t*-test, with *p*-value markers defined as follows: *** *p* ≤ 0.001, ** *p* ≤ 0.01, and * *p* ≤ 0.05.

To validate key RNA-Seq results, six representative DEGs, AR-PEL8 (JXL20A1288), AR-HSP70-17 (JXL20A0419), AR-PSBP (JXL14A1865), AR-CRD1 (JXL15A1736), AR-HO1 (JXL5A0019), and AR-DXS (JXL9A0582), were selected from the 16 identified DEGs in *A. roxburghii* for RT-qPCR validation (Table S2). These genes represent diverse functional categories, including stress response (AR-HSP70-17), photosynthesis (AR-PSBP), chlorophyll biosynthesis (AR-CRD1), cell wall modification (AR-PEL8), heme catabolism (AR-HO1), and terpenoid biosynthesis (AR-DXS), collectively reflecting physiological and metabolic responses to the tested conditions. The expression patterns of these genes were consistent with the RNA-Seq results, as indicated by the correlation coefficients (Figure 6). These findings confirm the robustness of the RNA-Seq data and demonstrate strong agreement between RT-qPCR and transcriptome sequencing in quantifying both upregulated and downregulated gene expression.

## 4. Discussion

*A. roxburghii* seeds exhibit biological constraints, including low natural germination rates, high rates of seed abortion, and elevated seedling mortality [26]. These intrinsic limitations, combined with external pressures such as overharvesting and habitat destruction, have resulted in severe depletion of wild resources. In this context, the development of tissue culture propagation and artificial cultivation techniques is of strategic importance for the sustainable development of the *A. roxburghii* industry. The TD method significantly improves phenotypic traits of tissue-cultured seedlings, including plant height, rooting rate, and transplant survival, by restoring photosynthetic autotrophic capacity [27,28].

This study employed integrated multiomics analysis, leveraging gene–metabolite association networks, to systematically unravel the metabolic advantages of the TD method over conventional CK approaches, thereby providing a theoretical framework for optimizing tissue culture systems. Furthermore, precise modulation of environmental parameters such as light intensity, photoperiod, and CO_2_ concentration enables targeted regulation of secondary metabolic pathways, which holds promise for enhancing the medicinal value of cultivated plants.

### 4.1. Comparative Study on Quality Differences Between TD and CK Methods for A. roxburghii

The closed environment of conventional tissue culture (CK) systems, characterized by high humidity, restricted CO_2_ availability, and suboptimal light, creates a heterotrophic growth niche that impairs the development of robust photosynthetic machinery and functional root systems. These constraints not only produce weak, etiolated plantlets but also compromise their post-transplant acclimation ability, leading to low field survival rates, as documented in prior studies [29].

In contrast, the sugar-free tissue culture (TD) system represents a paradigm shift by utilizing atmospheric CO_2_ as the primary carbon source instead of exogenous sucrose, forcing plants to transition to photoautotrophy [30]. This approach represents an important biotechnological tool for plant research, providing a platform for investigating plant growth and metabolic pathways, while also supporting plant propagation, genetic improvement, and production applications [31].

Our findings of enhanced rooting efficiency, transplant survival, and secondary metabolite accumulation in TD-grown *A. roxburghii* align with this developmental framework. The increased flavonoid and polysaccharide levels likely reflect strategic carbon reallocation toward stress-resistant compounds, supported by elevated antioxidant enzyme activity and improved cell membrane stability under drought stress, as observed in this study. These results highlight how cultivation strategy shapes plant phenotypic plasticity and metabolic resilience, with significant implications for commercial propagation where transplant success and consistent secondary metabolite production are critical economic factors [32,33].

### 4.2. Metabolite Profile Differences Between CK and TD Systems of A. roxburghii

In this study, the results demonstrate that the TD system markedly reshapes the metabolic network of *A. roxburghii*. Specifically, it downregulates the overall synthesis of amino acids and alkaloids while modulating the accumulation of secondary metabolites such as flavonoids and terpenoids. This metabolic reprogramming provides a molecular basis for the superior growth performance observed, including increased plant height, higher rooting rate, and improved transplant survival, and suggests potential shifts in medicinal component profiles. The identification of trans-zeatin, a natural cytokinin and plant growth regulator, among the top significantly accumulated DAMs (MEDP1808) in the TD system strongly suggests enhanced hormonal regulation and cellular differentiation activities, which likely contribute to the improved rooting efficiency and overall transplant robustness observed in TD-grown plantlets [34]. For example, compounds such as the terpenoids patrinoside and polyphyllin B, the flavonoids rotenone and icaritin, and organic acids such as citric acid and 3-furoic acid exhibited group-specific accumulation patterns [35].

These findings indicate that the TD system does not uniformly suppress secondary metabolism but instead induces selective metabolic reconfiguration. One possible explanation is that replacing exogenous sucrose with CO_2_-driven autotrophy alleviates sucrose-mediated feedback inhibition of photosynthesis and redirects carbon flux [36,37]. Reduced demand for nitrogen assimilation under autotrophic conditions may contribute to decreased amino acid biosynthesis. Concurrently, altered metabolic balance and enhanced photosynthetic efficiency may favor the synthesis of specific defensive or specialized metabolites, including certain flavonoids and terpenoids, which may be associated with improved stress tolerance. These metabolite profile differences provide a biochemical basis for the enhanced physiological performance (e.g., greater plant height and rooting rate) of TD seedlings and suggest the potential to manipulate bioactive compound accumulation to improve the medicinal quality of *A. roxburghii* under controlled tissue culture conditions.

### 4.3. Differential Gene Expression Profiling Between Two Tissue Culture Systems

Further transcriptomic analysis confirmed that the two tissue culture systems differentially influence the quality of *A. roxburghii* and provided additional insight into the underlying regulatory networks. A total of 416 DEGs were identified in the TD group, with a strong bias toward downregulation (372 genes). This widespread transcriptional downregulation likely reflects a transition to a more efficient and targeted metabolic state under photoautotrophic (TD) conditions, as energy and resources are redirected from non-essential or heterotrophy-specific processes toward those that support robust autotrophic growth [38]. These genes were significantly enriched in functional categories related to cell wall organization, extracellular and apoplastic processes, and photosynthesis [39]. This enrichment pattern is consistent with the observed superior growth phenotypes, including enhanced rooting and transplant survival, suggesting that the TD system promotes a developmental state favorable for autotrophic growth and stress acclimation.

Core photosynthesis-related genes were downregulated in TD plantlets, suggesting optimized photosynthetic efficiency via transcriptional reprogramming. Simultaneously, upregulation of genes associated with cell wall modification and stress response underpinned enhanced rooting ability and improved transplant survival. KEGG pathway enrichment identified DEGs associated with the MAPK signaling pathway, indicating activation of stress-adaptive signaling cascades [40]. Additionally, the enrichment of DEGs in the “other glycan degradation” pathway points to a remodeling of cell-wall-associated carbohydrate metabolism during the transition to photoautotrophy, which may facilitate cell wall loosening and restructuring, supporting more active cell expansion, tissue differentiation, and improved nutrient assimilation during TD cultivation [41]. Collectively, these results demonstrate that TD induces a coordinated shift in gene expression that prioritizes metabolic efficiency and structural resilience, thereby providing a molecular basis for improved seedling quality.

Core transcriptional differences were characterized by the identification of 416 DEGs, including marked downregulation of key genes involved in photosynthesis and chlorophyll biosynthesis (e.g., AR-PSBP, AR-CRD1, AR-HO1, and AR-DXS) in the TD group, reflecting optimized photosynthetic efficiency and reallocation of carbon flux under elevated CO_2_ autotrophic conditions [42]. Concurrently, genes related to cell wall structure and stress response (e.g., AR-PEL8 and AR-HSP70-17) were upregulated under TD conditions, providing molecular support for improved root development and transplant adaptability [43]. Together, these findings indicate that the autotrophic culture system systematically regulates pathways associated with photosynthesis, cell structural remodeling, and stress adaptation, thereby providing a transcriptional basis for enhanced growth performance and resilience of tissue-cultured plantlets [44,45]. However, this transcriptomic study was conducted at a single time point, and the dynamic regulatory changes during the transition from heterotrophic to photoautotrophic growth remain unclear. Future research employing time-series transcriptomics combined with functional validation of key candidate genes, such as those involved in the MAPK pathway, would be essential to delineate the causal regulatory network and further optimize the TD system for quality control.

## 5. Conclusions

This multi-omics study reveals that the TD system markedly enhances *A. roxburghii* seedling quality. TD plantlets show greater height, higher rooting and better transplant survival rates than CK. Transcriptomic analysis identifies 416 DEGs enriched in cell wall organization, apoplast and photosynthesis, with 16 key genes linked to growth and metabolism. Metabolomic profiling uncovers 502 DAMs, reflecting altered amino acid, alkaloid, flavonoid and terpenoid levels. Integrated analyses disclose coordinated gene–metabolite interactions, indicating TD improves seedling quality by regulating photosynthetic efficiency, carbon metabolism and secondary metabolite biosynthesis, offering a scientific basis for optimizing tissue culture production.

## Figures and Tables

**Table 1:** Comparative analysis of phenotypic traits in *A. roxburghii* plantlets cultured under different in vitro conditions.

Treatment	Rate of Survival (%)	Plant Height/cm	Rooting Rate/%	Growth Condition
Traditional tissue culture (CK)	79.41 ± 1.43 ^b^	4.87 ± 0.19 ^b^	65.67 ± 2.85 ^b^	Partial plants exhibited retarded growth, weak vigor, and reduced leaf size.
Sugar-free tissue culture (TD)	95.27 ± 2.11 ^a^	5.53 ± 0.21 ^a^	87.35 ± 2.43 ^a^	The plants exhibited robust and rapid growth with full and uniformly margined leaves.

Note: Different lowercase letters in the table indicate significant differences (*p* ≤ 0.05). SEM: standard error of the mean, quantifying the precision of the sample mean as an estimate of the population mean.

## Data Availability

The data presented in this study are available upon request.

## Supplementary Materials

The following supporting information can be downloaded at: https://www.mdpi.com/article/10.3390/metabo16060374/s1, Supplementary Table S1. RT-qPCR primers for 6 key candidate genes. Supplemental Table S2. Identification and functional annotation of differentially expressed genes of *Anoectochilus roxburghii* plantlets under different cultivation regimes. Supplementary Table S3. Widely Untargeted Metabolomics-Based Metabolite Profiling of *Anoectochilus roxburghii* Plantlets Under Different Cultivation Regimes. Supplementary Table S4. GO Functional Enrichment Analysis and Statistical Analysis of Differential Genes in *Anoectochilus roxburghii*. Supplementary Table S5. Differential Metabolites Screening of *Anoectochilus roxburghii* Plantlets Under Different Cultivation Regimes. Supplementary Table S6. Screening of key genes involved in flavonoid and terpenoid biosynthesis pathways in *A. roxburghii*.
